# Expanding the Phenotypic Spectrum of *PMPCB* ‐Related Disease: Long‐Term Survival with Childhood‐Onset Ataxia

**DOI:** 10.1002/mdc3.70921

**Published:** 2026-11-10

**Authors:** Ioannis Livanos, Christina Votsi, Marios Tomazou, Hermione Ugolini, Henry Houlden, Menelaos Pipis, Eleni Zamba Papanicolaou, Kyproula Christodoulou

**Affiliations:** ^1^ Neurogenetics Department The Cyprus Institute of Neurology and Genetics Nicosia Cyprus; ^2^ The Cyprus Institute of Neurology & Genetics, Member of the European Reference Network‐Rare Neurological Diseases (ERN‐RND) Tübingen Germany; ^3^ Genomics Core Facility The Cyprus Institute of Neurology and Genetics Nicosia Cyprus; ^4^ Bioinformatics Department The Cyprus Institute of Neurology and Genetics Nicosia Cyprus; ^5^ UCL Long Read Sequencing Facility UCL Queen Square Institute of Neurology London UK; ^6^ Department of Neurodegenerative Diseases UCL Queen Square Institute of Neurology London UK; ^7^ Department of Neuromuscular Diseases UCL Queen Square Institute of Neurology London UK; ^8^ Neuropathology Department The Cyprus Institute of Neurology & Genetics Nicosia Cyprus; ^9^ Neuroepidemiology Department The Cyprus Institute of Neurology & Genetics Nicosia Cyprus

**Keywords:** infantile‐onset ataxia, long‐read sequencing, MPP, Nanopore, *PMPCB*

*PMPCB*‐related disease is a rare mitochondrial disorder caused by biallelic variants affecting the catalytic β‐subunit of mitochondrial processing peptidase (MPP). MPP is a heterodimer consisting of the catalytic β‐subunit (PMPCB) and the non‐catalytic α‐subunit (PMPCA) that cleaves mitochondrial targeting presequences.^1^, ^2^ Previously reported individuals with PMPCB deficiency exhibited an infancy‐onset Leigh‐like disease with cerebellar atrophy that often resulted in early childhood death.^3^ Subsequent studies broadened the clinical spectrum, reporting patients surviving into adolescence and adulthood.^4^, ^5^, ^6^ Here, we describe a non‐consanguineous Cypriot family with two siblings in their 50s diagnosed with early‐onset cerebellar ataxia, further expanding the genotypic and clinical spectrum of *PMPCB*‐related disease.

The affected individuals tested negative for most ataxia‐associated repeat expansions. Whole‐exome sequencing was performed more than a decade ago, before the initial characterization of *PMPCB*‐related disease,^3^ and did not yield a genetic diagnosis. Detailed methodologies on whole‐genome long‐read sequencing, read processing, and analysis are provided in the Supplementary Methods.

Two siblings were evaluated at the clinic decades ago with early‐onset ataxia symptoms. The proband (II:1), a 58‐year‐old woman, at age 8 months, had a sudden regression with an episode of febrile convulsions. Early motor milestones were achieved, and she was able to walk with a walker at 9 months old. However, she achieved independent ambulation at age 11 and exhibited a clearly ataxic gait. Her gait ataxia has been slowly progressive over the years, and she currently exhibits a markedly ataxic gait with recurrent falls. The patient exhibited a scanning speech since early childhood and required specialized education throughout her childhood, likely due to concurrent learning difficulties and intellectual disability. Over time, additional neurological features appeared, including an evolving upper motor neuron syndrome with brisker reflexes in her mid‐30s and extensor plantar signs in her early 50s. Although her reflexes were initially attenuated and there was reduced vibration sensation to the ankle, nerve conduction studies showed no evidence of a large fiber polyneuropathy. She showed consistent evidence of partial complex ophthalmoplegia (unilateral or bilateral failure of eye abduction) in early adulthood and at different times in her late 40s/early 50s. She also showed mild symmetrical eyelid ptosis. Her 50‐year‐old brother (II:3) achieved early gross motor milestones but crawled until age 6 years and walked independently at the age of 7 years with significant gait unsteadiness. Like his older sister, he had abnormal speech from early childhood and was occasionally enrolled in specialized curricula throughout his schooling. The affected brother also exhibited multidirectional gaze‐evoked nystagmus, and his gait unsteadiness has been progressively worsening. Neither sibling showed evidence of hearing loss, retinopathy, or optic atrophy, but visual evoked potentials in the brother showed right optic pathway dysfunction. Serum lactate was within normal limits in both siblings. CT brain of the brother, done following a minor injury, showed a hypoplastic cerebellum and vermis (Fig. 1A). The phenotypic features of both siblings are presented in Table S1.

**Figure 1:**
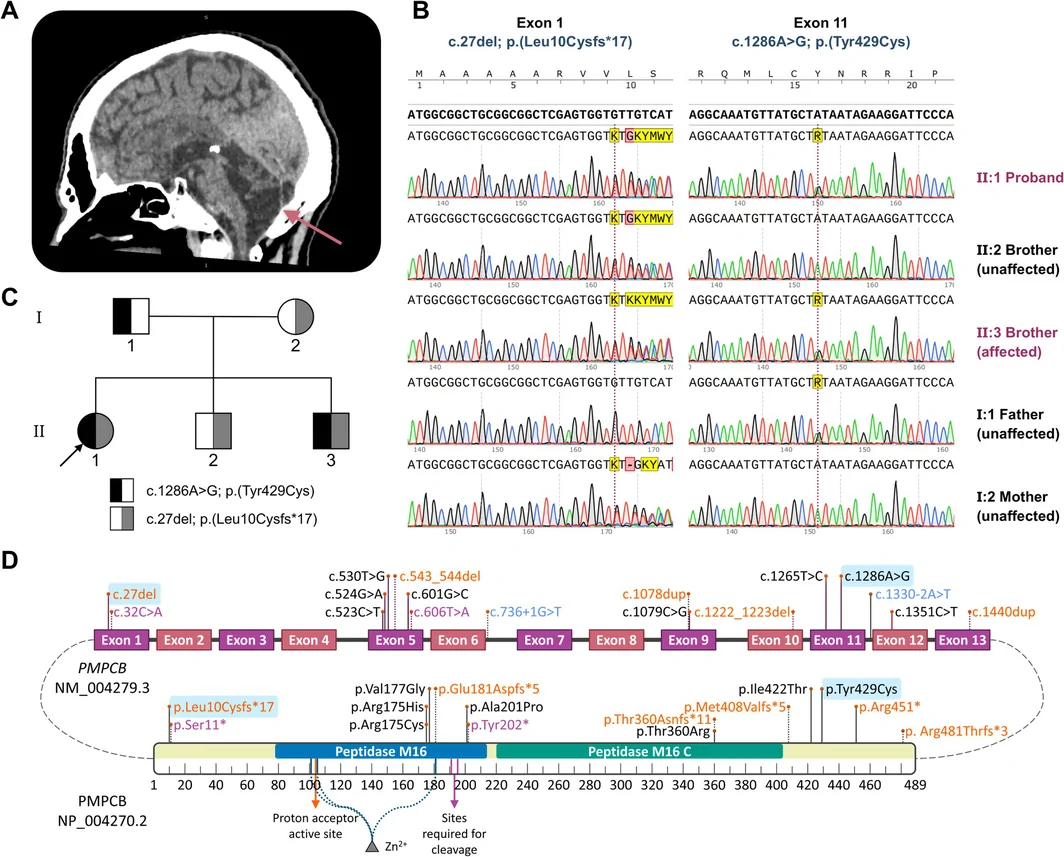
Genetic characterization of *PMPCB* variants. (A) Sagittal midline image from CT brain showing hypoplastic cerebellum and cerebellar vermis. (B) Sanger validation of c.27del and c.1286A>G. (C) Family pedigree with variant segregation. (D) Variants identified in this study are highlighted in light blue. Solid lines show variants reported in the literature, while dotted lines denote pathogenic or likely pathogenic variants (<50 bp) found exclusively in ClinVar.

Long‐read sequencing identified two *PMPCB* variants, a missense variant (c.1286A>G) and a novel single‐nucleotide deletion (c.27del), separated by 14.4 kb (Fig. S1). Despite the limited read‐length N50 (7.2 kb), an 18.3‐kb haploblock was identified across this region, enabling haplotype phasing, confirming that both variants were *in trans* in the affected siblings (Fig. S2). These variants are either absent or extremely rare (<0.001%) in population databases (Table S2). Sanger sequencing validated the presence of the variants (Fig. 1B) and co‐segregation across the pedigree was established (Fig. 1C). The p.(Leu10Cysfs*17) variant introduces a premature stop codon early in the coding sequence and is therefore expected to result in loss‐of‐function (LOF) through nonsense‐mediated mRNA decay (NMD) (Fig. 1D). The missense variant p.(Tyr429Cys) impacts a highly conserved residue (Fig. S3) and is predicted to be deleterious by several in silico tools (Table S2). The c.27del and c.1286A>G variants, based on the ACMG criteria,^7^ are predicted to be pathogenic and likely pathogenic, respectively.

The first reported individuals with *PMPCB*‐related disease presented with infancy‐onset Leigh‐like symptoms and a notably high childhood mortality rate.^3^ A subsequent report described the first adult patient, a 39‐year‐old woman, with a comparatively milder phenotype.^4^ In contrast, the phenotype in this sibship is primarily an early‐onset cerebellar ataxia with some Leigh‐like features and, most importantly, survival into the sixth decade. Tyr429 is evolutionarily conserved across distantly related metazoan species, but it lies outside the catalytically active M16 domain. The milder phenotype observed here may indicate partial preservation of PMPCB function, particularly due to the C‐terminal location of the p.(Tyr429Cys) missense variant, which may allow residual MPP activity.

This study describes a novel early frameshift variant *in trans* with a missense variant. To our knowledge, including those described in this study, only 11 individuals with *PMPCB*‐related disease have been described in the literature to date.^3^, ^4^, ^5^, ^6^ The affected siblings represent the oldest surviving individuals described with biallelic *PMPCB* variants, demonstrating early‐onset ataxia with unusually long survival. Long‐read sequencing confirmed the allelic configuration of the compound heterozygous variants, resolving a decade‐long diagnostic challenge and highlighting its value in unresolved ataxia cases.

## Author Roles

(1) Research project: A. Conception, B. Organization. C. Execution; (2) Statistical analysis: A. Design, B. Execution, C. Review and critique; (3) Investigation: A. Experiments, B. Analysis; (4) Bioinformatic analysis: A. Design, B. Execution, C. Review and critique; (5) Clinical evaluation: A. Sample acquisition, B Patient evaluation; (6) Funding acquisition; (7) Manuscript Preparation: A. Writing of the first draft, B. Review and critique.

I.L.: 1, 2, 3, 4, 7.

C.V.: 1A, 1B, 2C, 7B.

M.T.: 4C, 7B.

H.U.: 3A, 7B.

H.H.: 6, 7B.

M.P.: 5B, 7B.

E.Z.P.: 5, 7B.

K.C.: 1A, 2C, 6, 7B.

## Disclosures

**Ethical Compliance Statement:** Written informed consent was obtained from all individuals participating in the study. Individuals were pseudonymized. The study was conducted in accordance with the guidelines of the Declaration of Helsinki and with the General Data Protection Regulation of the European Union (2016/679). This study has been approved by the National Bioethics Committee of Cyprus (ΕΕΒΚ/ΕΠ/2013/28). We confirm that we have read the Journal's position on issues involved in ethical publication and affirm that this work is consistent with those guidelines.

**Financial Disclosures and Conflicts of Interest:** The open access fees for this publication are covered by the CING. This study was funded by the Cyprus Telethon awarded to K.C. (Grant no. 73285). H.H. is supported by Parkinson's UK and the UK Dementia Research Institute through UK DRI Ltd, principally funded by the Medical Research Council. H.H. also thanks the Wellcome Trust (Award 221,951/Z/20/Z). Nothing to declare. Author disclosures are available in the Supporting Information.

## Supporting information


**Data S1.** ICMJE Disclosure Statement Form.

**Table S1.** Phenotypes of affected family members. A complete list of the phenotypic features of each affected sibling, along with associated HPO terms.
**Table S2.** Annotation and in silico pathogenicity assessment of *PMPCB* variants. Variant details, population frequencies, and predictions are summarized. The c.27del is absent from population databases, while c.1286A>G is observed at an extremely low allele frequency.
**Figure S1.** IGV visualization of phased *PMPCB* variants. Haplotype‐resolved long‐read alignments at the *PMPCB* locus. Reads are grouped and color‐coded by haplotype phasing, indicating that the two variants are located on different haplotypes, consistent with the compound heterozygous inheritance pattern.
**Figure S2.** IGV visualization of the haplotype block spanning *PMPCB*. Both variants fall within the same 18.3 kb phased set, which covers the majority of the NM_004279.3 transcript.
**Figure S3.** Multiple sequence alignment of PMPCB orthologous proteins. PMPCB is highly conserved across evolutionarily distant orthologs. Although Tyr429 lies outside the canonical peptidase M16 domain and towards the C‐terminus, the residue is invariant across phylogenetically diverse metazoan species.

## Data Availability

The data that support the findings of this study are available on request from the corresponding author upon reasonable request. The data are not publicly available due to privacy or ethical restrictions. The commands used for bioinformatic analyses are provided in the supplementary methods. This study did not generate any new code. All bioinformatic tools utilized in this study are publicly available on GitHub.
