# Supplementary material for: Expanding the Phenotypic Spectrum of PMPCB ‐Related Disease: Long‐Term Survival with Childhood‐Onset Ataxia
Source: Mov Disord Clin Pract. 2026 Nov 10:10.1002/mdc3.70921. Online ahead of print. doi: 10.1002/mdc3.70921 (PMC13752520; doi:10.1002/mdc3.70921)
Supplement: Supplementary file 2 [file MDC3-9999-0-s002.pdf]

Table of Contents

Supplementary Information 2

Supplementary Methods 2

Whole-genome long-read sequencing 2

Read processing and data analysis 2

Annotation and structural analysis 2

Nanopore sequencing 2

wf-alignment 3

wf-human-variation 3

Sniffles2 5

AnnotSV 5

TRGT 6

Supplementary Results 8

FGF14 expansion (SCA27B) 9

RFC1-related expansion (*RFC1*-related ataxia) 9

Variants surrounding *PMPCB* 10

AlphaFold-predicted PMPCB structure 11

Supplementary Tables 13

Supplementary Figures 15

Supplementary Information

Supplementary Methods

Whole-genome long-read sequencing

DNA fragment size was assessed using the Femto Pulse system (Agilent Technologies, Santa Clara, CA, USA). Light shearing was performed on the Megaruptor 3 (Diagenode, Liege, Belgium). Libraries were prepared using the Ligation Sequencing Kit V14 (SQK-LSK114) and sequenced on R10.4.1 flow cells on the PromethION24 (Oxford Nanopore Technologies, Oxford, UK).

Read processing and data analysis

Input reads were aligned against the GRCh38 reference genome using the wf-alignment (v1.2.2) workflow, and variants were identified using the wf-human-variation (v2.6) workflow (ONT EPI2ME labs). Variant annotation and pathogenicity assessment were performed using eVai cloud-based interpreter (EnGenome, Pavia, Italy). Pathogenicity classification of variants was based on the ACMG/AMP 2015 guidelines. Furthermore, Sniffles2 (v2.6.2) was also utilized as a standalone tool, followed by AnnotSV (v3.4.6) to annotate the >20,000 identified SVs. Finally, TRGT (v4.1.0) with the STRchive repeat catalogue was employed to analyze >60 known medically relevant STR loci.

Annotation and structural analysis

Multiple sequence alignment (MSA) of UniProt PMPCB orthologs from distantly related taxa was performed using MAFFT (v7) to assess the evolutionary conservation of the p.(Tyr429Cys) variant. The potential effect on residue interactions surrounding the missense variant was evaluated using the AlphaFold-predicted PMPCB (O75439) structure. Variant pathogenicity predictions were performed using CADD (v1.7), MutationTaster2025, and VEP (r115). Variants were confirmed with Sanger sequencing (Fig. 1a).

Nanopore sequencing

Basecalling was carried out using the R10.4.1 flow cell and the v4.3.0 high-accuracy (HAC) basecalling model (dna_r10.4.1_e8.2_400bps_hac@v4.3.0).

wf-alignment

ONT EPI2ME labs developed the wf-alignment (v1.2.2) workflow, which internally utilizes minimap2 for alignment. FASTQ (Q ≥9) reads were aligned against a reference. Internally, wf-alignment invokes minimap2 using the default DNA preset. The reads were aligned to the UCSC GRCh38 analysis set reference genome, which excludes alternative loci. The wf-alignment command is shown below:

```
nextflow run epi2me-labs/wf-alignment \
  --fastq /data/fastq_pass \
  --references data/UCSC/hg38.analysisSet.fa \
  --out_dir /data/alignment_output \
  --igv true \
  --threads <threads> \
  --prefix sample_id \
  -profile standard
```

wf-human-variation

The wf-human-variation (v2.6) workflow analyzed the aligned BAM file generated by wf-alignment. The wf-human-variation workflow comprises several distinct sub-workflows: small-variant (SNVs and indels ≤ 50 bp) calling with Clair3, SV (≥ 30 bp) calling with Sniffles2, large CNV calling with Spectre, STR genotyping with Straglr, and phasing with WhatsHap. The basecaller override option was used to select the appropriate small-variant calling model for Clair3. Sniffles2 by default detects SVs of at least 30 bp. However, using the default preset, variants between 30 and 50 bp may be reported by both Sniffles2 and Clair3. To prevent overlap, Sniffles was configured to report SVs > 50 bp (--sniffles_args '--minsvlen 51'). To improve SV calling in repetitive regions, Sniffles2 supports the use of a tandem-repeat annotation file, specified via the '--tr_bed' option. The annotation file for the appropriate human reference genome is available through the Sniffles GitHub repository. Straglr, within wf-human-variation, interrogates 32 medically relevant STR loci, based on predefined criteria. The workflow annotates variants using SnpEff (supporting GRCh37 and GRCh38) and adds ClinVar annotations to the SNV calls. Annotations ('--annotation false') were disabled for the following reasons: (1) variant annotation and pathogenicity assessment were performed using the eVai cloud-based interpreter, and (2) embedding annotations within VCF files significantly increases file size.

```
nextflow run epi2me-labs/wf-human-variation \
  --bam /data/alignment/sample_id.sorted.aligned.bam \
  --ref /data/UCSC/hg38.analysisSet.fa \
  --snp --sv --cnv --str --phased \
  --sex XY \ # (XX or XY) passed to Straglr, if not specified, workflow infers
  --sniffles_args '--minsvlen 51' \
  --tr_bed /data/annotations/human_GRCh38.trf.bed \
  --bam_min_coverage 0 \
  --annotation false \ # [default: true]
  --igv true \
  --override_basecaller_cfg dna_r10.4.1_e8.2_400bps_hac@v4.3.0 \
  --out_dir /data/wf_human_variation/output \
  --sample_name sample_id \
  --threads <threads> \
  -profile standard
```

Sniffles2

Sniffles2 (v2.6.2) can detect all of the most recognized simple types of SVs, but it can also detect more complex variants. It can effectively manage the relatively high sequencing error rates present in LRS technologies, particularly the relatively high indel errors common in nanopore reads. For improved calling in repetitive regions, Sniffles2 supports the use of a TR annotation file, specified via the '--tandem-repeats' option. The annotation file is available through the Sniffles GitHub repository. Standalone Sniffles2 was used to generate more compact VCF outputs by omitting the read-name field. A chromosome-only SV analysis was executed here. The desired chromosomes were specified using the '--contig' option. Unlocalized and unplaced scaffolds were excluded from the analysis.

```
sniffles \
--input /data/sample_id.haplotagged.cram \
--vcf /data/sample_id_sniffles2.vcf \
--snf /data/sample_id_sniffles2.snf \
--reference /data/UCSC/hg38.analysisSet.fa \
--tandem-repeats /data/human_GRCh38_no_alt_analysis_set.trf.bed \
--phase \
--minsvlen 51 \ # [default: 50]
--threads <threads> \
--sample-id sample_id \
--contig chr1 --contig chr2 --contig chr3 --contig chr4 --contig chr5 --contig
chr6 --contig chr7 --contig chr8 --contig chr9 --contig chr10 --contig chr11 --
contig chr12 --contig chr13 --contig chr14 --contig chr15 --contig chr16 --contig
chr17 --contig chr18 --contig chr19 --contig chr20 --contig chr21 --contig chr22 -
-contig chrX --contig chrY # chrY omitted for females
```

AnnotSV

The VCF output files from the Sniffles2 analysis were annotated using AnnotSV (v3.4.6). SVs represent a substantial source of variability within the human genome. AnnotSV provides detailed SV annotations by compiling functional, regulatory, and clinical data. It uses Human Phenotype Ontology (HPO) terms, along with Exomiser and PhenoGenius, to prioritize genes that overlap with SVs based on the patient's phenotype. This HPO-based scoring method enables the identification of genes previously associated with disease and deprioritizes genes linked to phenotypes with little or no resemblance to the patient's phenotypic features.

Genes overlapped by SVs were annotated using Ensembl, as it contains a larger set of transcripts. AnnotSV evaluates pathogenicity and assigns a ranking score (class 1-5), supporting clinical interpretation. Variants classified as benign or likely benign were excluded using the '-rankFiltering 3-5,NA' option, retaining only variants of uncertain significance, likely

pathogenic, pathogenic, and unclassified variants. Finally, the lowest possible allele frequency, 0.1%, was used, as we are interested only in rare diseases.

```
AnnotSV \
  -SVinputFile /data/sample_id_sniffles2.vcf \
  -outputFile /data/sample_id_annotated.tsv \
  -vcf 1 \
  -benignAF 0.001 \ # [default: 0.01]
  -genomeBuild GRCh38 \
  -SVminSize 51 \ # [default: 50]
  -rankFiltering 3-5,NA \ # [default: 1-5,NA]
  -annotationMode both \
  -tx ENSEMBL \ # [default: RefSeq]
  -REreport 1 \
  -hpo HP:0000001,HP:0000002,HP:0000003 # [HPO IDs describing the phenotype]
```

```
perl /data/knotAnnotSV2XL.pl \ # Excel file creation
  --annotSVfile /data/sample_id_annotated.tsv \
  --configFile /data/config_AnnotSV.yaml \
  --outDir /data/sample_id \
  --genomeBuild GRCh38 \
  --geneCountThreshold 40
```

TRGT

Pathogenic repeat expansions often surpass the read length of short-read platforms, and their repetitive nature further complicates alignment and variant calling. In contrast, long reads can effectively cover most medically relevant STRs, allowing comprehensive variant analysis in these low-complexity regions of the genome. Orthogonal validation may still be necessary for diagnostic purposes.

TRGT examines repeat loci based on a repeat catalogue supplied by the user. In this analysis, a BED file containing 73 medically relevant STR loci was obtained from the STRchive GitHub repository. TRGT requires a reference genome (FASTA), an input alignment file (BAM or CRAM), and a repeat definition file in BED format.

```
trgt genotype \
  --genome /data/UCSC/hg38.analysisSet.fa \
  --reads /data/sample_id.haplotagged.cram \
  --repeats /data/STRchive-disease-loci.hg38.TRGT.bed \
  --output-prefix /data/sample_id.TRGT \
  --karyotype XY \ # (XX or XY) [default: XX]
  --threads <threads> \
  --preset wgs # wgs or targeted [default: wgs]
```

```
bcftools sort -Oz \
  -o /data/sample_id.TRGT.sorted.vcf.gz \
  /data/sample_id.TRGT.vcf.gz

bcftools index \
  /data/sample_id.TRGT.sorted.vcf.gz
```

```
samtools sort \
  -o /data/sample_id.TRGT.spanning.sorted.bam \
  /data/sample_id.TRGT.spanning.bam

samtools index \
  /data/sample_id.TRGT.spanning.sorted.bam
```

Supplementary Results

In addition to the established repeat expansion causes of spinocerebellar ataxia, recently identified loci were also assessed. The affected individuals tested negative for SCA1, SCA2, SCA3, SCA6, SCA7, SCA8, SCA10, SCA12, SCA17, SCA27B, SCA36, DRPLA, Friedreich's ataxia (FRDA), and the pathogenic *RFC1*-related repeat expansions. These repeat loci were assessed using fluorescent long-range PCR (LR-PCR), followed by fragment analysis by capillary electrophoresis, automated electrophoresis, and agarose gel electrophoresis, confirming the absence of pathogenic expansions. Following long-read sequencing, these repeat loci were re-evaluated with TRGT, which also did not identify pathogenic expansions. No pathogenic expansions were detected across >70 known STR loci, including those not associated with ataxia. Representative TRGT results for both affected family members are shown below for *FGF14* and *RFC1* repeat expansions.

FGF14 expansion (SCA27B)

Individual	*FGF14* Allele 1 (length in bp)	*FGF14* Allele 1 (motif count – pure repeats)	*FGF14* Allele 2 (length in bp)	*FGF14* Allele 2 (motif count – pure repeats)
Father I:1	44	9	65	16
Mother I:2	50	11	247	76
**Affected Daughter II:1**	**41**	**8**	**289**	**86**
Non-affected Son II:2	50	11	68	17
**Affected Son II:3**	**68**	**17**	**349**	**111**

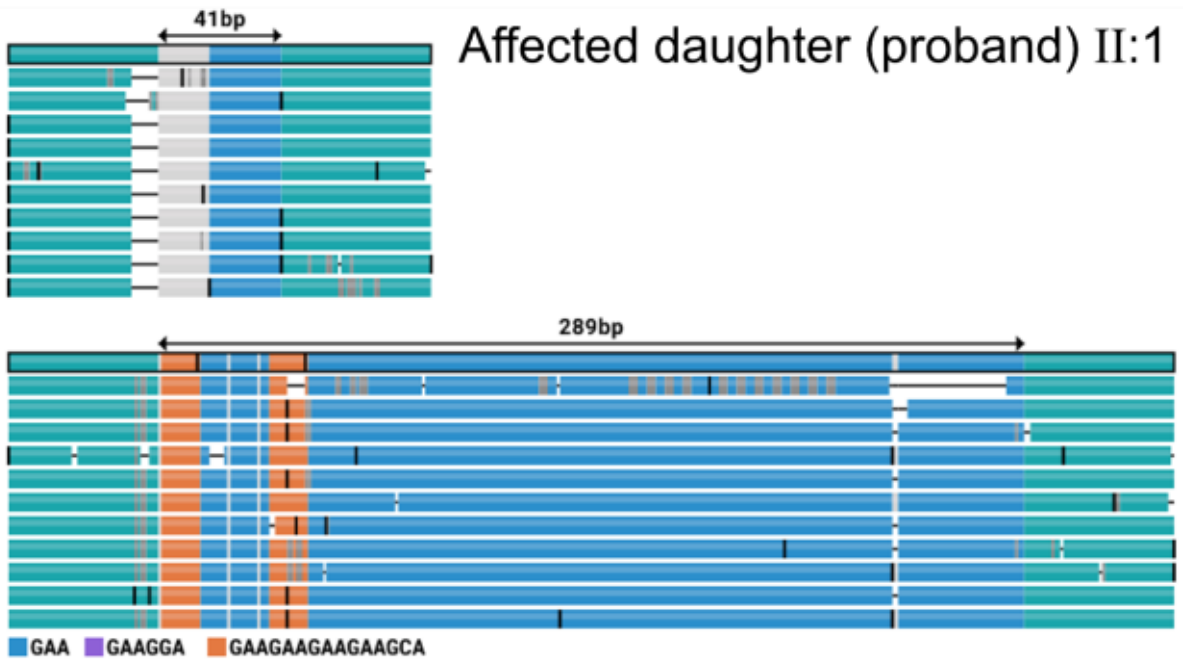

Affected daughter (proband) II:1

Affected son II:3

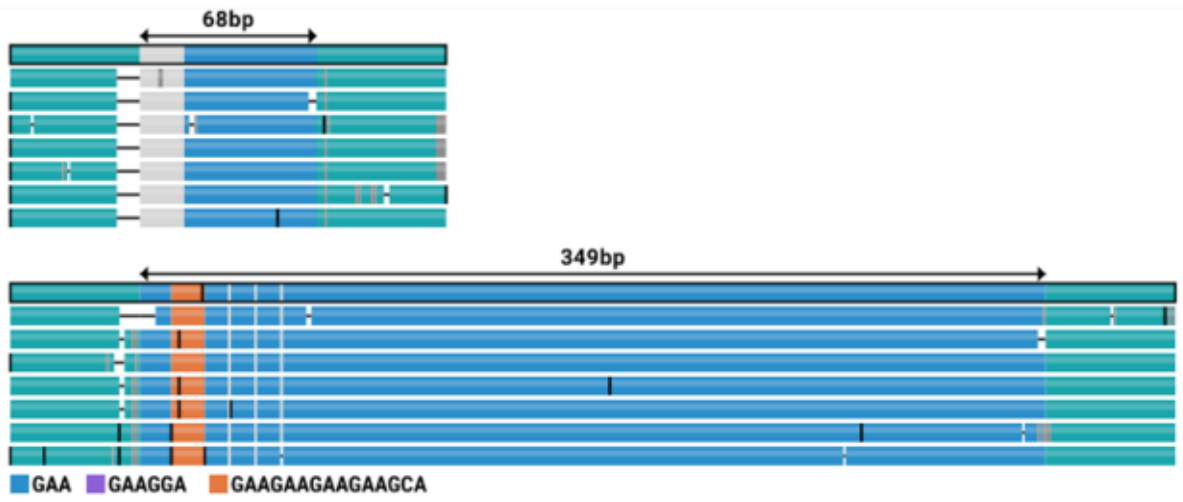

RFC1-related expansion (*RFC1*-related ataxia)

Individual	*RFC1* Allele 1 (length in bp)	*RFC1* Allele 1 (motif count – pure repeats)	*RFC1* Allele 2 (length in bp)	*RFC1* Allele 2 (motif count – pure repeats)
Father I:1	482	64	577	112
Mother I:2	54	9	54	10
**Affected Daughter II:1**	**54**	**10**	**494**	**98**
Non-affected Son II:2	54	10	478	73
**Affected Son II:3**	**54**	**10**	**489**	**93**

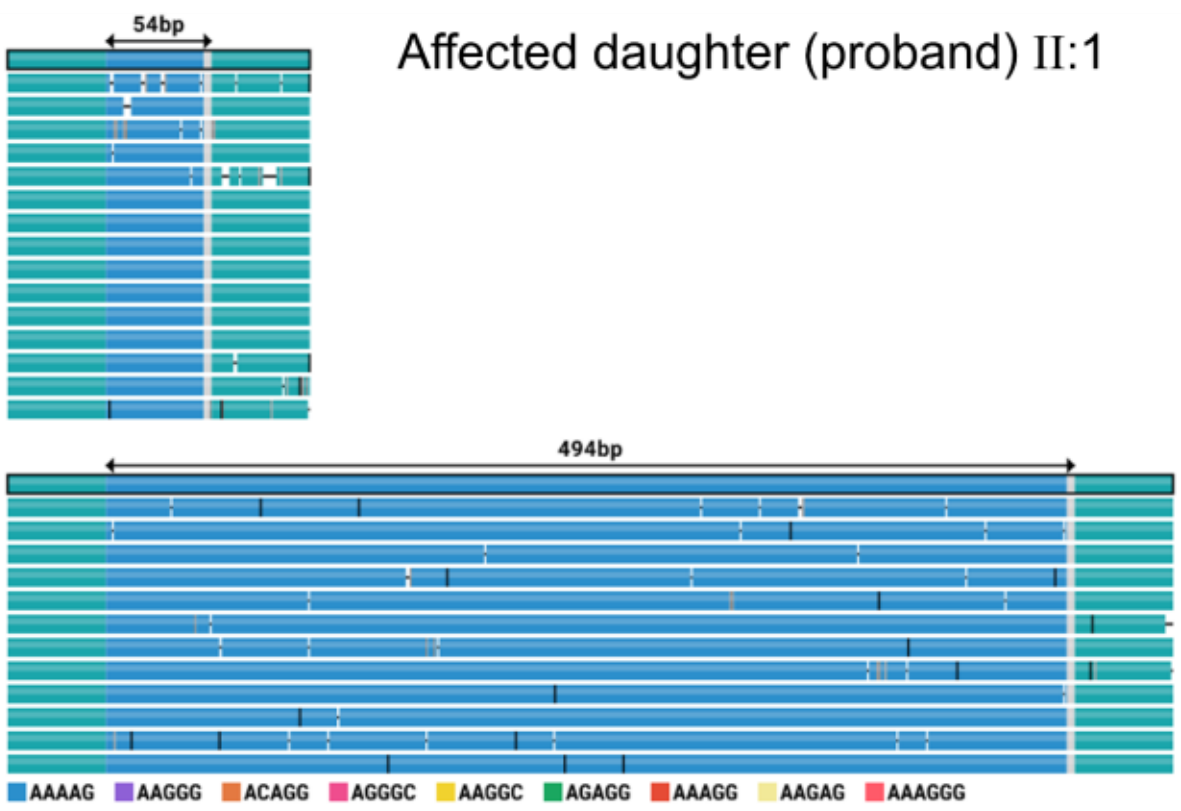

Affected daughter (proband) II:1

Affected son II:3

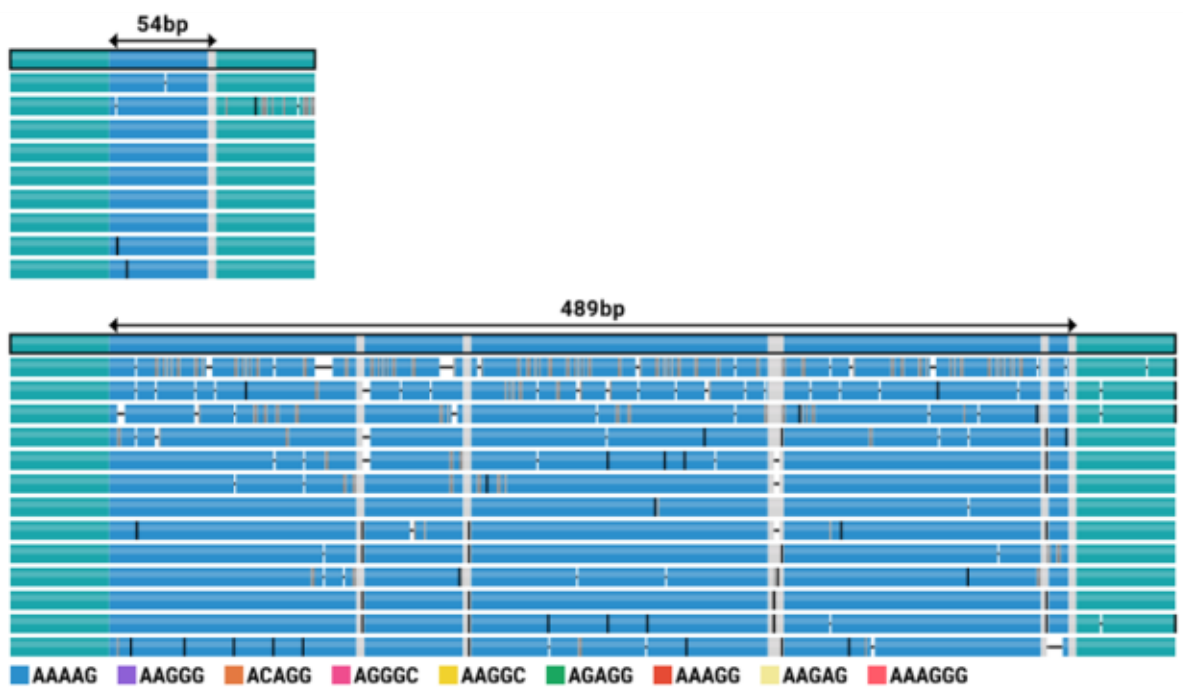

Variants surrounding *PMPCB*

II:1 Affected Daughter (proband)

Position	Reference	Alternate	Quality*	Gene	Location
chr7:103293511	C	T	9.52	-	Intergenic
chr7:103296080	G	T	29.92	-	Intergenic
chr7:103297485	**TG**	**T**	**17.18**	***PMPCB***	**Exon**
chr7:103307593	T	C	30.45	*PMPCB*	Intron
chr7:103311853	**A**	**G**	**26.24**	***PMPCB***	**Exon**
chr7:103315169	AT	A	5.97	*DNAJC2*	Intron
chr7:103316549	A	C	30.42	*DNAJC2*	Intron

II:3 Affected Son

Position	Reference	Alternate	Quality*	Gene	Location
chr7:103293511	C	T	27.6	-	Intergenic
chr7:103296080	G	T	28.54	-	Intergenic
chr7:103297485	**TG**	**T**	**19.45**	***PMPCB***	**Exon**
chr7:103307593	T	C	30.41	*PMPCB*	Intron
chr7:103311853	**A**	**G**	**25.97**	***PMPCB***	**Exon**
chr7:103315169	AT	A	5.04	*DNAJC2*	Intron
chr7:103316549	A	C	30.24	*DNAJC2*	Intron

Table entries were taken from the Clair3 VCF outputs for each affected individual.

*Variants with very low quality scores (QUAL ≤5) were excluded from display.

AlphaFold-predicted PMPCB structure

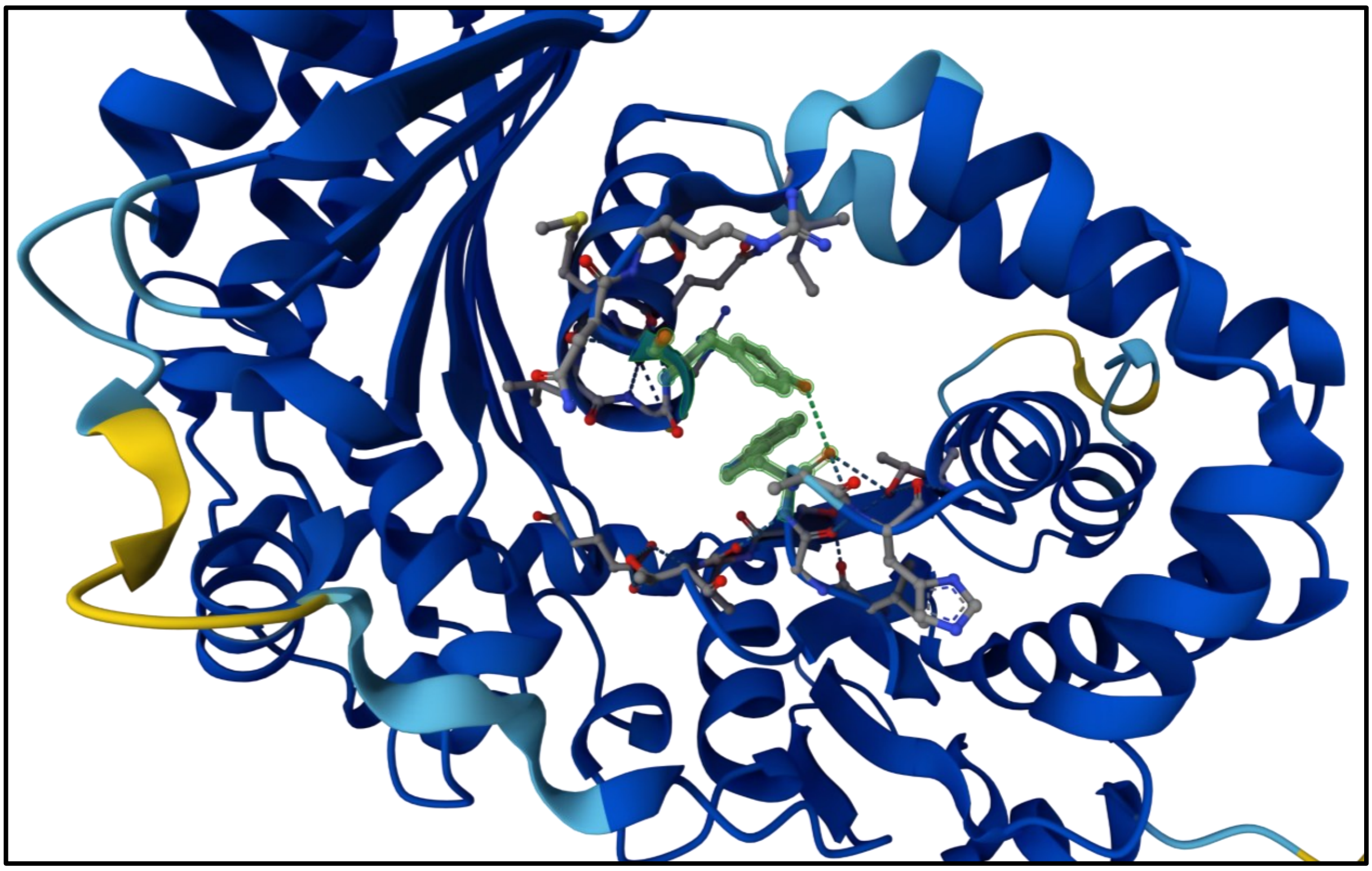

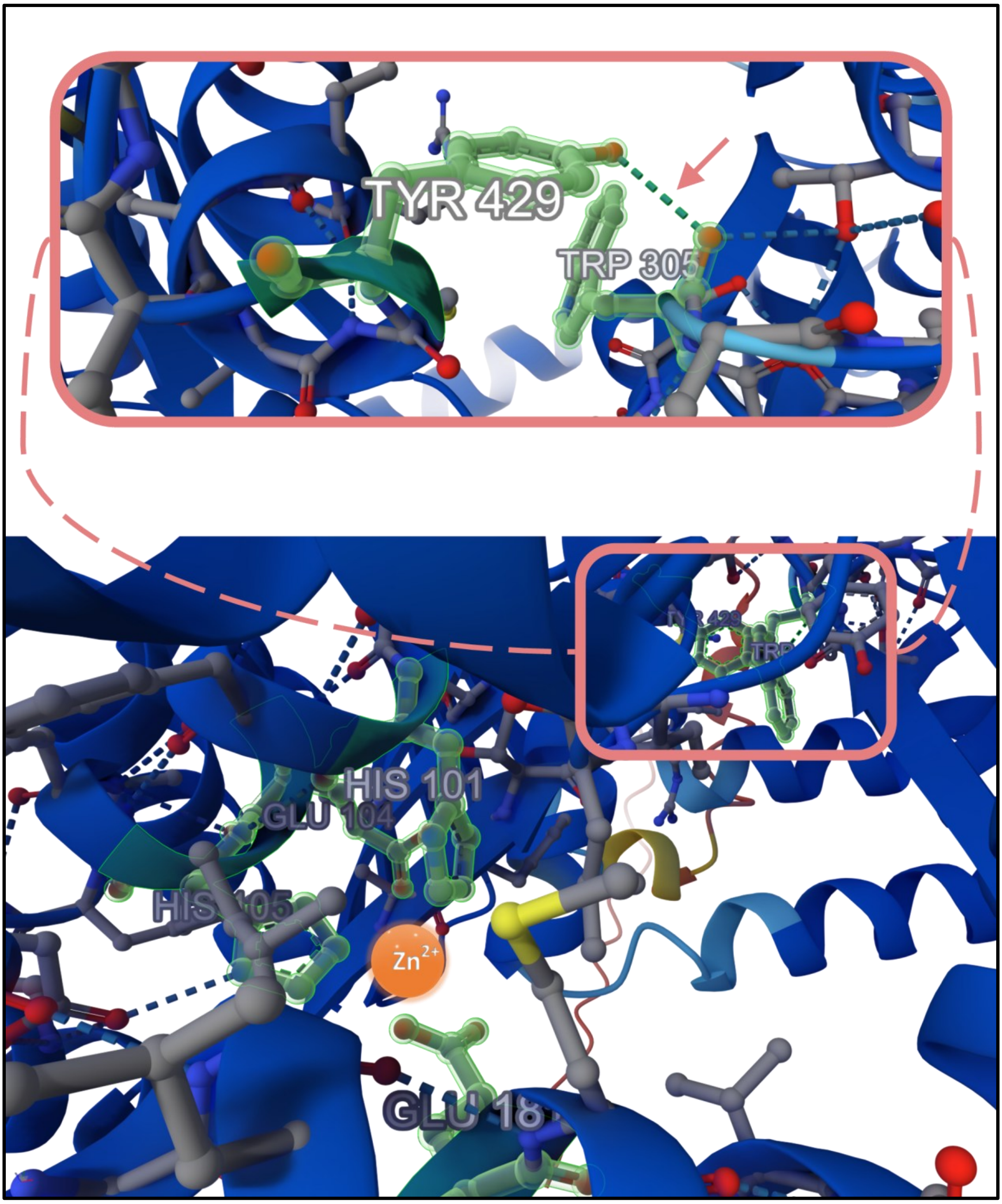

The AlphaFold PMPCB protein model showed that Tyr429 is located distal to the catalytic site. Tyr429 is predicted to be in proximity to Trp305. Its phenolic hydroxyl group is positioned near the backbone carbonyl of Trp305 at a predicted distance of ~3.0 Å, potentially forming a hydrogen bond that may contribute to local structural organization. A cysteine substitution in this context would disrupt the interaction, potentially compromising the local structural stability. Finally, the spatial position of Tyr429 relative to the catalytic site is shown.

Supplementary Tables

HPO term	Affected sibling (proband) II:1	Affected sibling II:3
HP:0000182	Abnormality of lingual movement	Abnormality of lingual movement
HP:0000634	Impaired ocular abduction	-
HP:0000648	-	Optic atrophy
HP:0000763	Sensory neuropathy	-
HP:0001249	Intellectual disability	Intellectual disability
HP:0001251	Ataxia	Ataxia
HP:0001260	Dysarthria	Dysarthria
HP:0001320	-	Cerebellar vermis hypoplasia
HP:0001321	-	Cerebellar hypoplasia
HP:0001348	Brisk reflexes	-
HP:0001488	Bilateral ptosis	-
HP:0002066	Gait ataxia	Gait ataxia
HP:0002078	Truncal ataxia	Truncal ataxia
HP:0002080	Intention tremor	-
HP:0002136	Broad-based gait	Broad-based gait
HP:0002168	Scanning speech	Scanning speech
HP:0002373	Febrile seizure	-
HP:0002464	Spastic dysarthria	-
HP:0002493	Upper motor neuron dysfunction	-
HP:0002522	Areflexia of lower limbs	-
HP:0003487	Babinski sign	Babinski sign
HP:0006938	Decreased vibration sense at ankles	-
HP:0007979	-	Gaze-evoked horizontal nystagmus
HP:0011477	-	Upbeat nystagmus
HP:0020035	Lower limb dysmetria	Lower limb dysmetria
HP:0031936	Delayed ability to walk	Delayed ability to walk
HP:0033229	-	Brachioradialis areflexia
HP:0033257	-	Delayed ability to walk with support
HP:0033683	Jaw hyperreflexia	-

Table S1: Phenotypes of affected family members. A complete list of the phenotypic features of each affected sibling, along with associated HPO terms.

<table>
<tr><td>Variant information</td><td></td><td></td></tr>
<tr><td>DNA change</td><td>g.103297486del</td><td>g.103311853A>G</td></tr>
<tr><td>cDNA change</td><td>c.27del</td><td>c.1286A>G</td></tr>
<tr><td>Protein change</td><td>p.(Leu10Cysfs*17)</td><td>p.(Tyr429Cys)</td></tr>
<tr><td>dbSNP</td><td>-</td><td>rs2484901209</td></tr>
<tr><td>Consequence</td><td>Frameshift variant</td><td>Missense variant</td></tr>
<tr><td>Exon</td><td>1</td><td>11</td></tr>
<tr><td>MANE transcript</td><td colspan="2">NM_004279.3</td></tr>
<tr><td>MANE protein</td><td colspan="2">NP_004270.2</td></tr>
<tr><td>Population frequencies</td><td></td><td></td></tr>
<tr><td>gnomAD: AF (Eur.)</td><td>-</td><td>0.000005935</td></tr>
<tr><td>All of Us: AF (Eur.)</td><td>-</td><td>0.000004</td></tr>
<tr><td>In silico predictions</td><td></td><td></td></tr>
<tr><td>CADD PHRED</td><td>18.37</td><td>28.5</td></tr>
<tr><td>MutationTaster</td><td>NMD</td><td>87|13 (deleterious)</td></tr>
<tr><td>AlphaMissense (VEP)</td><td>-</td><td>0.411 (ambiguous)</td></tr>
<tr><td>REVEL (VEP)</td><td>-</td><td>0.583 (uncertain)</td></tr>
<tr><td>ClinPred (VEP)</td><td>-</td><td>0.997 (pathogenic)</td></tr>
<tr><td>EVE score (VEP)</td><td>-</td><td>0.673 (pathogenic)</td></tr>
<tr><td>PaPI (eVai)</td><td>0.892 (damaging)</td><td>1.000 (damaging)</td></tr>
<tr><td>ACMG</td><td></td><td></td></tr>
<tr><td>Criteria</td><td>PVS1 PM2 PM3 PP1 PP4</td><td>PM2 PM3 PP1 PP3 PP4</td></tr>
<tr><td>Classification</td><td>Pathogenic</td><td>Likely pathogenic</td></tr>
</table>

Table S2: Annotation and *in silico* pathogenicity assessment of *PMPCB* variants. Variant details, population frequencies, and predictions are summarized. The c.27del is absent from population databases, while c.1286A>G is observed at an extremely low allele frequency.

Supplementary Figures

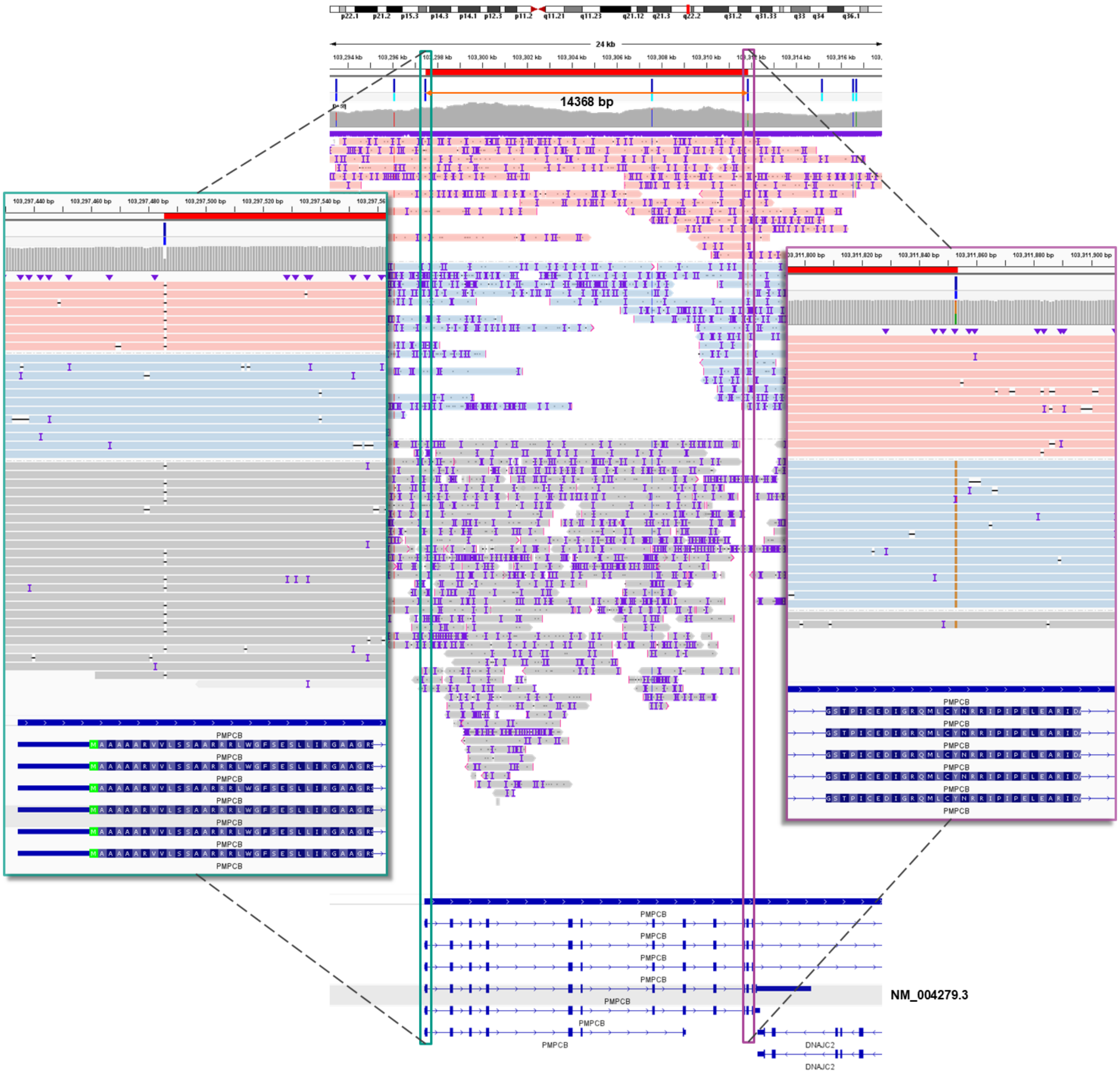

Figure S1: IGV visualization of phased *PMPCB* variants. Haplotype-resolved long-read alignments at the *PMPCB* locus. Reads are grouped and color-coded by haplotype phasing, indicating that the two variants are located on opposite haplotypes, consistent with the compound heterozygous inheritance pattern.

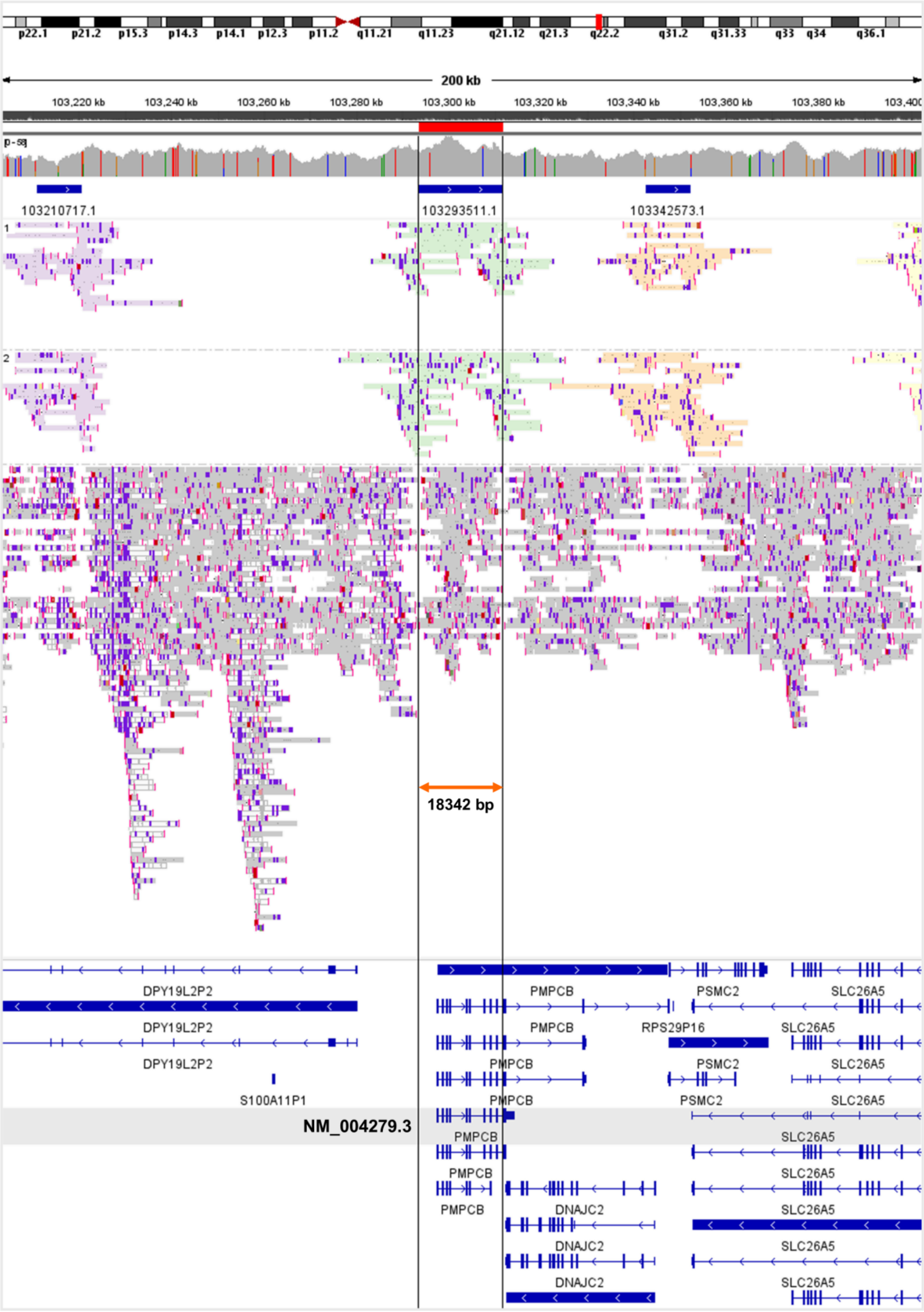

Figure S2: IGV visualization of the haplotype block spanning *PMPCB*. Both variants fall within the same 18.3 kb phased set, which covers the majority of the NM_004279.3 transcript.

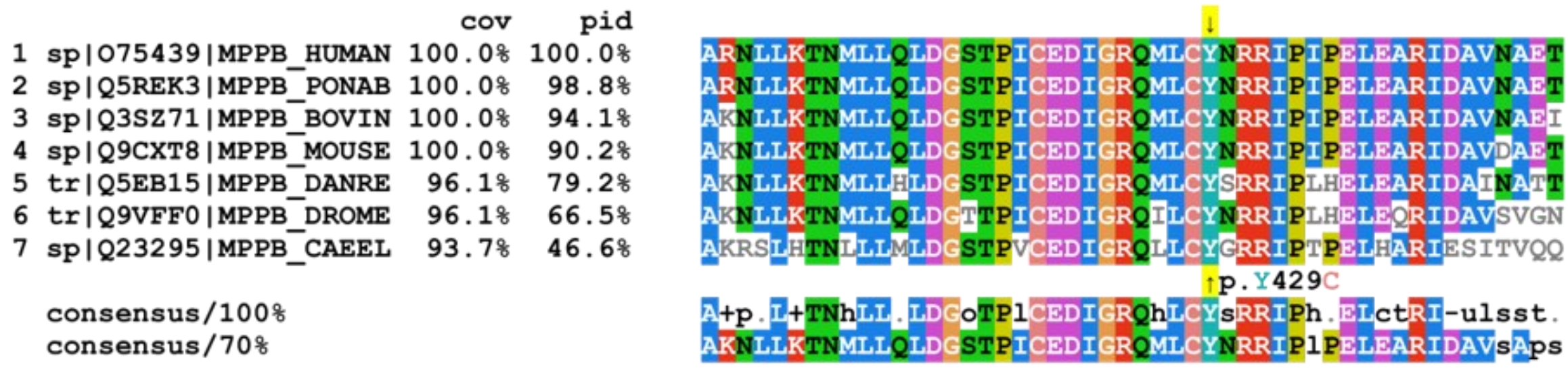

Figure S3: Multiple sequence alignment of PMPCB orthologous proteins. PMPCB is highly conserved across evolutionarily distant orthologs. Although Tyr429 lies outside the canonical peptidase M16 domain and towards the C-terminus, the residue is invariant across phylogenetically diverse metazoan species.
